# Myelin Antigens and Antimyelin Antibodies

**DOI:** 10.3390/antib7010002

**Published:** 2018-01-02

**Authors:** Fredrick J. Seil

**Affiliations:** Department of Neurology, Oregon Health & Science University, Portland, OR 97239, USA; seilf@comcast.net

**Keywords:** myelin, experimental allergic encephalomyelitis, myelin basic protein, immunoglobulin, myelin oligodendrocyte glycoprotein, multiple sclerosis

## Abstract

The purpose of this review is to provide an historical perspective on studies of serum derived antimyelin antibodies. Antimyelin antibodies can be defined by their action on myelinating organotypic nervous system tissue cultures and include demyelinating antibodies, which have destructive effects on myelin when applied to already myelinated cultures, and myelination inhibiting antibodies, which prevent myelin formation when applied to cultures prior to myelination. Myelin antigens were evaluated in animal studies for their ability to induce experimental allergic encephalomyelitis, an inflammatory demyelinating disease, and correlated with the induction of antimyelin antibodies. As tissue culture demyelinating activity was also found in sera from some patients with multiple sclerosis, a human inflammatory demyelinating disease, studies were undertaken to characterize the nature of the demyelinating factors.

## 1. Introduction

Over half a century ago, Bornstein and Appel [1] found that sera from rabbits with experimental allergic encephalomyelitis (EAE) induced by inoculation with whole central nervous system (CNS) tissue and Freund’s complete adjuvant (FCA) demyelinated rat organotypic cerebellar cultures. The sera were applied directly as a component of the culture nutrient medium. Demyelination was complement dependent because heating the sera to 56 °C abolished the demyelinating activity, which was restored by the addition of fresh guinea pig serum. The demyelination was specific for CNS myelin as rat dorsal root ganglia cultures, which contained peripheral nervous system (PNS) myelin, were unaffected. Remyelination of the cerebellar cultures followed replacement of the sera from animals with EAE induced by inoculation with whole CNS (anti-CNS sera) with normal nutrient medium. In a subsequent study, Appel and Bornstein [2] reported that demyelinating activity was found in the immunoglobulin G2 (IgG2) fraction of anti-CNS sera and was abolished by absorption with homologous or heterologous brain tissue, but not with other tissues such as lung, liver, kidney, or red blood cells. With the use of immunofluorescent techniques, globulins in demyelinating sera were localized on myelin sheaths of cerebellar cultures, which was confirmed in a later study with immunoperoxidase methods [3]. The presence of demyelinating activity in the IgG2 fraction of anti-CNS sera was also noted by Lebar et al. [4], who did not find similar activity in IgG1 or IgM serum fractions.

When Bornstein and Raine [5] applied anti-CNS sera to CNS cultures at explantation, prior to myelin formation, the maturation of oligodendrocytes, the CNS myelin forming cells, and CNS myelination were inhibited. The inhibition of myelination was complement dependent and reversible, was specific for oligodendrocytes and CNS myelin, and was obtained at far lower concentrations than required for demyelination. Because of the last attribute, myelination inhibition appeared to the authors to be a more sensitive and reliable index of antimyelin activity than demyelination. Given that the demyelinating and myelination inhibiting factors appeared to be antibodies, they became collectively known as “antimyelin antibodies.” As EAE could also be induced by sensitization with CNS myelin or myelin components, a series of studies was initiated to investigate which myelin components were responsible for the induction of antimyelin antibodies, and whether or not the induction of these antibodies correlated with the induction of EAE.

Soon after the initial description of demyelinating activity in sera from animals with EAE induced by inoculation with whole CNS and FCA, Bornstein [6] reported finding similar demyelinating activity in sera from 68% of human subjects with active multiple sclerosis (MS), the most common of the human demyelinating diseases. The demyelination was reversible upon removal of the patient sera. Most normal human sera did not demyelinate CNS cultures. These results suggested that humoral factors, possibly antibodies, might have a pathogenetic role in MS and led to further investigation of these factors.

## 2. Myelin

Prior to further discussion of demyelination, it would be of value to briefly review some of the salient morphological and biochemical features of normal myelin. More detailed descriptions can be found in other sources [7,8].

**Figure 1:**
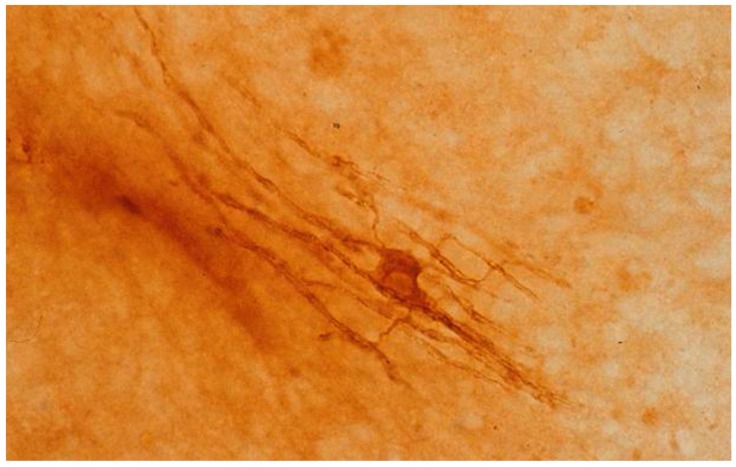
Oligodendrocyte from a mouse cerebellar culture after 15 days in vitro, with slender cytoplasmic processes projecting to contiguous myelin sheaths. The culture was reacted with antibody to human myelin basic protein and processed by the peroxidase-antiperoxidase method.

As already noted, the CNS myelin forming cell is the oligodendrocyte, while PNS myelin is formed by Schwann cells. An oligodendrocyte contributes cytoplasmic processes to the formation of multiple internodes of central myelin (Figure 1), while a Schwann cell forms a single internode of peripheral myelin. Injury of an oligodendrocyte results in the loss of a plurality of myelinated internodes, while the destruction of a Schwann cell results in the loss of one internode. 

The nodes of Ranvier are unmyelinated gaps between internodes of myelinated axonal segments. Both CNS and PNS myelin are formed by the spiral wrapping of cytoplasmic processes of the respective myelin forming cells around central or peripheral axons, followed by extrusion of the cytoplasm to allow apposition of the inner cytoplasmic membranes. These apposed membranes constitute the osmiophilic interperiod or major dense lines evident on ultrastructural examination. The less densely staining intraperiod or minor dense lines are formed by apposition of the outer cytoplasmic membranes of the myelin forming cells. The ultrastructural appearance of a myelin sheath is thus one of alternating dark major and minor dense lines with light intervals between. It is thought that the dense lines contain the protein components of the myelin sheath, while the lucent intervals represent bimolecular leaflets of lipid. The external component of the myelin sheath is an outer cytoplasmic membrane. In the PNS, there is also a basement membrane, which is a component of the Schwann cell.

CNS and PNS myelin differ in chemical composition. While both varieties have a high lipid:protein ratio and a similar proportion of cholesterol, CNS myelin has more galactolipid (cerebroside and sulfatide) and less phospholipid [8]. The major CNS myelin protein is the proteolipid protein, which constitutes approximately one-half of the protein in central sheaths [9]. One-third of the CNS myelin proteins consists of myelin basic protein, a cathode-migrating protein. A lesser fraction (20%) of CNS myelin protein is an acidic proteolipid protein (Wolfgram protein), whereas glycoprotein is a very small constituent of CNS myelin [9,10]. The major component of PNS myelin is a glycoprotein, P_0_ [11]. Two basic protein fractions in PNS myelin are P_1_, which is identical to CNS myelin basic protein, and P_2_, which is unique to peripheral myelin. The three proteins, P_0_, P_1_, and P_2_, constitute about 70% of the PNS myelin proteins, while proteolipid and Wolfgram proteins are absent in PNS myelin [12].

## 3. EAE and Anti-MBP Antibodies

The first attempt to correlate EAE induction with the presence of antibodies to specific CNS myelin components was made by Lumsden [13], who applied sera from animals inoculated with a diffusible encephalitogenic myelin peptide to organotypic CNS cultures. The failure of the sera to demyelinate the cultures was attributed by Lumsden to a lack of “antigenicity” of his peptide. The first established CNS myelin protein capable of inducing EAE was myelin basic protein (MBP) [14,15]. We subsequently tested the ability of sera from guinea pigs with EAE inoculated with the whole MBP molecule plus FCA to demyelinate mouse cerebellar cultures [16]. As a positive control, we also applied sera from whole CNS sensitized guinea pigs to myelinated cultures. In order to ensure that adequate levels of antibody were attained by MBP sensitization, we initially inoculated guinea pigs with MBP plus incomplete Freund’s adjuvant followed by a challenge dose of MBP plus FCA, which produced high titers of antibody to MBP in three gamma globulin classes. None of the sera from animals with EAE induced by sensitization with MBP or from the hyperimmunized animals demyelinated CNS cultures. The cultures were demyelinated by sera from whole CNS sensitized animals, as initially reported by Bornstein and Appel [1], but none of these sera had detectable levels of antibody to MBP. 

We followed this study with a series of investigations of various aspects of the EAE to anti-MBP antibody relationship. Such studies were always controlled by a comparison with the effects of sera from whole CNS sensitized animals. Initially, we evaluated myelination inhibition by sera from guinea pigs inoculated with heterologous (bovine) MBP plus FCA compared to sera from whole CNS plus FCA sensitized guinea pigs [17]. Most sera from the former, containing high levels of antibody to MBP, failed to inhibit the myelination of cerebellar cultures, while most of the latter sera, with low levels of anti-MBP antibody, inhibited myelin formation. In a following study [18], EAE was induced in subhuman primates by inoculation with either whole CNS plus FCA or MBP plus FCA. None of the sera from MBP sensitized animals inhibited the myelination of cerebellar cultures, while all of the sera from animals with EAE induced by whole CNS were positive for myelination inhibition. Similar results were obtained with sera from Lewis rats sensitized with FCA plus either guinea pig whole CNS tissue or guinea pig MBP [19], as the former prevented myelination in vitro, while the latter did not inhibit the myelination of cerebellar cultures. As a further extension of these studies, myelination inhibiting properties of sera from rabbits sensitized with bovine CNS tissue and sensitized or hyperimmunized with MBP from five different species, including bovine, monkey, human, guinea pig, and rabbit, were compared [20]. All of the rabbits inoculated with whole CNS and FCA developed EAE and all or their sera inhibited myelination, in the absence of detectable levels of the precipitating antibody to MBP. The rabbits sensitized or hyperimmunized with MBP developed a spectrum of possible combinations of EAE and the precipitating antibody. Serum from one of nine such animals was positive for myelination inhibition and this animal had neither EAE nor the anti-MBP antibody, whereas sera from the remaining rabbits, including those with EAE and high levels of the anti-MBP antibody, did not inhibit myelination. In a final study with MBP [21], the majority of sera from guinea pigs inoculated at intervals with MBP plus incomplete Freund’s adjuvant and followed by inoculation with whole CNS plus FCA inhibited myelination in cerebellar cultures, although the animals did not develop EAE. This indicates that protection of guinea pigs with MBP prevented EAE induction by whole CNS, but did not prevent the induction of myelination inhibiting antibodies. Collectively, these studies demonstrated a complete dissociation of serum demyelinating and myelination inhibiting activity, the induction of EAE, and the formation of the anti-MBP antibody.

## 4. Other CNS Myelin Antigens

As it was evident that MBP, the major encephalitogenic myelin protein, did not evoke antimyelin antibodies, other myelin antigens were investigated as possible agents. Dubois-Dalcq et al. [22] reported that sera from rabbits inoculated with FCA plus cerebroside, a lipid component of CNS myelin that did not induce EAE, demyelinated CNS cultures. The demyelination was not restricted to CNS myelin, as some peripherally myelinated fibers in spinal cord plus dorsal root ganglia cultures were also affected. The CNS demyelinating activity of sera from cerebroside sensitized rabbits was confirmed by Fry et al. [23], who additionally showed that these sera inhibited the myelination of CNS cultures. Antimyelin activity was abolished by absorption with cerebroside. The peripherally demyelinating properties of rabbit anticerebroside antisera were duplicated by Saida et al. [24], providing a contrast to the reported specificity of antisera to whole CNS tissue for activity against CNS myelin [1,5,25]. Antimyelin activity appeared to be restricted to sera from rabbits sensitized with cerebroside, for sera from Lewis rats inoculated with cerebroside did not inhibit the myelination of cerebellar cultures [19] and sera from guinea pigs inoculated with cerebroside did not demyelinate CNS cultures [4]. Hruby et al. [26] showed that the myelination of cerebellar cultures was inhibited by sera from rabbits sensitized with synthetic galactocerebroside (GC), ruling out any possibility of contamination with other myelin components. Similar results were not obtained with rabbit antisera directed against glucocerebroside, indicating that the antimyelin activity was specific to anti-GC sera.

Demyelinating activity was found by Lebar et al. [27] in sera from guinea pigs inoculated with an antigen designated as “M2” found in a “myelin-like” fraction isolated from CNS myelin. The myelin-like fraction did not contain cerebroside or GM1 ganglioside and did not crossreact with MBP or the myelin proteolipid protein. M2 was not present in peripheral myelin and was later determined to be a glycoprotein component of the oligodendrocyte membrane [28]. Its ability to induce EAE was not determined. 

Since MBP appeared to be localized in the major dense (interperiod) line of CNS myelin [29] and thus not available at the surface of the myelin sheaths, it was of interest to determine the capability of antimyelin antibody induction by myelin-associated glycoprotein (MAG), a minor component of CNS myelin that did not induce EAE, but was localized at the myelin membrane surface [30,31]. Sera from rabbits inoculated with purified MAG neither demyelinated nor inhibited myelination in CNS cultures [32]. The major CNS myelin protein, proteolipid protein (PLP), was reported to induce a chronic form of EAE in rabbits [33,34]. Rabbit antisera to PLP also did not demyelinate or inhibit the myelination of CNS cultures, as determined in two separate laboratories [35,36]. An autoimmune demyelinating disease was described in rabbits by inoculation with gangliosides, glycolipid components of myelin and neuronal membranes [37,38,39]. We found no demyelinating or myelination inhibiting activity in spinal cord plus dorsal root ganglia cultures exposed to four rabbit high titer antisera directed to GM1, a major ganglioside [40].

When CNS myelin is extracted with chloroform-methanol, PLP, almost all of the MBP and most of the lipids are removed [41]. The remaining chloroform-methanol insoluble protein (CMIP) fraction contains a host of low and high molecular weight proteins, including the glycoproteins, among which are MAG, M2, and the myelin oligodendrocyte glycoprotein (MOG), which is a minor myelin protein localized at the external surfaces of myelin sheaths and oligodendrocyte membranes [42,43,44,45]. Rabbits inoculated with the CMIP fraction and FCA did not develop clinically manifested EAE, but did have occasional focal mononuclear infiltrates in perivascular spaces, sometimes with spread into the surrounding CNS parenchyma, characteristic of histological features of EAE [46]. Antisera to the CMIP fraction inhibited the myelination of cerebellar cultures and demyelinated centrally myelinated fibers in spinal cord-dorsal root ganglia cultures, while sparing peripherally myelinated fibers. The demyelination and myelination inhibition were rapidly reversed upon the removal of anti-CMIP sera from the cultures. On electron microscopic examination [47], oligodendrocyte maturation was not inhibited, contrary to the effect of anti-CNS antiserum [5]. It appeared that anti-CMIP sera interfered with glial-axonal interactions to inhibit myelin formation rather than with oligodendrocyte maturation. With regard to MOG, which induces both acute and chronic EAE [48,49,50], the application of a monoclonal anti-MOG antibody to reaggregating myelinated brain cell cultures caused a reversible complement dependent demyelination [51]. Myelination inhibition was not tested and the antibody was not applied to cultures with PNS myelin. As MOG is present in the CMIP fraction of myelin, it is likely that the anti-MOG antibody would inhibit myelination and its antimyelin activity would be specific for CNS myelin. It is also probable that MOG and M2 [28] are the same glycoproteins. 

## 5. PNS Myelin Antigens and EAN

A PNS equivalent of EAE is experimental allergic neuritis (EAN), which can be induced with the inoculation of FCA plus whole PNS tissue [52] or with the P_2_ fraction of PNS myelin [53,54]. Yonezawa et al. [55] reported the demyelination of PNS cultures by rabbit and guinea pig antisera directed against whole PNS tissue, a finding confirmed in later studies [56,57,58]. We additionally reported the inhibition of peripheral myelination by anti-PNS sera [58]. In these studies, CNS myelinated fibers were also demyelinated and CNS myelination was inhibited by antisera to PNS myelin. Uyemura et al. [59] found demyelination only in PNS cultures exposed to sera from rabbits inoculated with FCA and a myelin fraction derived from bovine spinal roots. Mithen et al. [60] applied serum from a goat sensitized with P_2_ derived from rabbit sciatic nerve to myelinated dorsal root ganglia cultures and found no demyelination and also no binding to the surfaces of Schwann cells. We [58] found no demyelination or myelination inhibition of either PNS or CNS myelin in mouse spinal cord-dorsal root ganglia cultures by rabbit anti-P_2_ sera with detectable levels of antibody to P_2_. The neuritogenic protein, like its encephalitogenic counterpart, MBP, did not induce demyelinating or myelination inhibiting factors. Presumably, these antibodies are directed against some other antigen in peripheral myelin.

## 6. Significance of Antimyelin Antibodies

A summary of the various antigens evaluated for their ability to induce antimyelin antibodies is presented in Table 1. Also indicated in the table is whether or not the antigens induced experimental demyelinating disease. It is evident from this table that the induction of EAE and EAN can be completely dissociated from the induction of antimyelin antibodies, raising the question of the relevance of these antibodies to the pathogenesis of disease. Although such antibodies may not be essential to disease induction, they may augment demyelination. Raine et al. [61] inoculated guinea pigs with FCA plus either (a) whole CNS white matter or (b) MBP or (c) MBP and GC or (d) MBP and total myelin lipids. Sensitization to whole white matter or MBP provoked clinically similar EAE, but histologically, both inflammatory and demyelinative lesions were seen in EAE induced by whole white matter, whereas only inflammatory lesions were seen in MBP induced EAE. When MBP was injected in combination with either GC or total myelin lipids, EAE with both inflammatory and demyelinative lesions was evident. Injection of FCA plus either GC or total myelin lipids alone induced neither EAE nor histopathological changes. The lipid haptens appeared to have an augmenting effect on MBP, and the speculation was advanced that MBP triggered the T-cell component of the immune response in EAE and that B-cell secreted factors evoked by GC or other lipid haptens are necessary for demyelination.

Bourdette et al. [62] initially gave guinea pigs a suboptimal transfer (insufficient to transfer EAE) of lymphocytes sensitized to MBP, which was then followed by the inoculation of FCA and (a) MBP or (b) chicken brain alone or (c) MBP plus chicken brain or myelin. Chicken MPB is not encephalitogenic in guinea pigs [63], so the chicken brain or myelin served as a source of all of the non-MBP components of CNS myelin, including lipids and glycoproteins. Myelination inhibiting activity in CNS cultures by sera from these guinea pigs was determined and correlated with the degree of histologically graded demyelination of spinal cord and brain sections [62]. Myelination inhibiting antibodies and histologically moderate to severe demyelination were found only in sera from animals receiving both MBP and chicken brain or myelin, and not with MBP or chicken brain alone. These findings were consistent with the concept of augmentation of demyelination by antibodies directed against nonencephalitogenic components of CNS myelin.

The notion of a combined action of cellular and antibody mechanisms to produce inflammatory demyelinating lesions in EAE was further supported by the finding that MOG, which induced EAE with both inflammatory and demyelinative components, elicited a T-cell mediated immune response and a B-cell secreted demyelinating antibody response [48,49,50,51]. A correlation was found between in vivo demyelinating activity in guinea pigs with chronic EAE and titers of antibody to MOG [64]. In another study [65], demyelination was greatly augmented by the intravenous injection of a monoclonal anti-MOG antibody during the induction of EAE in rats by the transfer of MBP sensitized T-cells. The combined action of cellular and antibody mechanisms is not an invariable requirement for demyelination in EAE, however, as extensive demyelination can occur in some species or strains and in some circumstances without augmentation by antibodies. In one such study [66] in which chronic relapsing EAE was induced in SJL/J mice by passive transfer with MBP sensitized T-cells, a considerable degree of demyelination occurred, even during initial episodes, in the absence of antibodies that bind to myelin. Antimyelin antibodies have also been reported to opsonize myelin for phagocytosis by macrophages [67].

## 7. Human Demyelinating Disorders

Only limited studies have been done on antimyelin antibodies in human PNS demyelinating diseases. Cook et al. [68] found that sera from 84% of patients with idiopathic polyneuritis (Guillain-Barré syndrome), in which peripheral nerves and nerve roots are segmentally demyelinated, showed demyelinating activity when applied to mouse dorsal root ganglia cultures. Demyelination was complement dependent and was blocked by prior exposure of sera to the guinea pig sciatic nerve. The demyelinating activity was associated with serum IgG and IgM fractions. Sera from some patients with idiopathic polyneuritis also demyelinated CNS cultures. Similar results were obtained by Dubois-Dalcq et al. [69], who additionally noted that patterns of myelin breakdown were similar to those seen in the demyelination of tissue cultures by anti-PNS antisera.

After Bornstein’s [6] initial study in which CNS cultures were demyelinated by 68% of sera from patients with active MS, Bornstein and Hummelgard [70] reported on serum demyelinating activity in an expanded series of human subjects with MS. They found demyelinating activity in 64% of sera from patients with definitely active disease, 41% of sera from patients without clearly evident disease activity at the time of serum collection, 11% of sera from patients without disease activity, and 7% of sera from normal subjects. When the course of disease was considered, 71% of sera from patients with active exacerbating and remitting MS demyelinated CNS cultures compared to 48% with a chronic progressive course. In more recently reported studies, Ulrich and Lardi [71] found demyelinating activity in 36% of patients with active disease and in 6% with inactive disease. We [72] described the demyelination of CNS cultures by 40% of subjects with active MS compared with 5% of sera collected during stationary periods. Sera from only 14% of patients with progressive MS were positive for demyelination. Dau et al. [73] reported no sera positive for in vitro demyelinating activity in any of seven patients with chronic progressive MS before or after plasmapheresis. Bradbury et al. [74] found demyelinating activity in 74% of sera from patients with MS, in 68% of sera with various neurological diseases other than MS, and in 22% of sera from normal subjects. The occurrence of demyelinating activity in a significant number of sera from patients with other neurological diseases, especially amyotrophic lateral sclerosis (ALS), had been previously reported [6,75].

Sera from patients with MS specifically demyelinated CNS cultures and did not affect peripherally myelinated fibers [25]. Unexpectedly, demyelinating MS sera did not inhibit myelination of spinal cord and cerebellar cultures [71,72]. Demyelinating activity was removed by absorption with brain tissue [6] and a nonmyelin tissue pellet that included oligodendrocytes, but not with purified myelin [76]. No immunostained myelin was evident when demyelinating MS sera were applied to CNS cultures and fixed and treated with peroxidase conjugated anti-human globulin [3]. Depletion of MS sera of all γ-globulin did not decrease their demyelinating capability and isolated Ig fractions from most MS sera did not demyelinate CNS cultures [77,78]. These findings indicate that demyelinating activity in most MS sera is associated with some serum factor other than γ-globulin. This, plus the lack of specificity of serum demyelinating factors for MS, led to some questions as to the significance of these factors for the pathogenesis of MS.

Of interest, therefore, was a report [79] of the detection of anti-MOG antibodies bound to disintegrating myelin sheaths in acute lesions from three patients with MS. The pattern of demyelination and the anti-MOG antibody binding were identical to those seen in marmosets with EAE induced by the inoculation of FCA and MOG [79,80]. Thus, MOG could be a target for antibody mediated demyelination in some cases of MS [81]. However, serum demyelinating factors in most cases of MS do not appear to be antibodies [3,77,78], so that B-cell demyelination may be restricted to a small subset of MS patients, lending support to the notion that MS is an autoimmune inflammatory demyelinating disease with heterogeneous pathology and pathogenesis [81,82,83,84,85].

More recently, Elliott et al. [86] found complement dependent demyelinating activity when purified IgG from about 30% of MS patients was applied to myelinated CNS cultures and evaluated with a sensitive bioassay for myelin reduction. Demyelinating activity was not observed in cultures exposed to IgG from patients with other neurological diseases or normal controls. IgG preparations with demyelinating activity contained antibodies that bound to fully differentiated oligodendrocytes and to their contiguous myelin sheaths, and not to oligodendrocyte precursors. Demyelinating antibodies were detected more frequently in patients with relapsing-remitting MS than in those with a chronic progressive course. Plasma exchange significantly reduced demyelinating IgG activity in three MS patients. Adsorption of MS patient derived IgG with MOG did not reduce the demyelinative capability of the IgG preparations, indicating that MOG is not the dominant target for demyelinating antibodies in the patients studied. These results reinforced the concept of a heterogeneous pathogenesis for MS.

Anti-MOG antibodies have been reported in pediatric inflammatory demyelinative disorders [87,88] and in a subset of patients with neuromyelitis optica [89]. The primary antigenic target in the latter disease is aquaporin-4, which is present in astrocytes [90]. Anti-MOG antibodies were present in cases of neuromyelitis optica negative for anti-aquaporin-4 antibodies.

## 8. Conclusions

Although demyelination can occur under some circumstances in the experimental animal disease, EAE, in the absence of antimyelin antibodies, it is clear that antimyelin antibodies augment demyelination. The inflammatory component of the disease represents a T-cell mediated immune response, but the demyelinative component is enhanced by B-cell secreted antibodies. The role of antimyelin antibodies in human demyelinating disorders is less clear. That antibodies contribute to the pathogenesis of some cases of MS is indicated by clinical improvement after plasma exchange in some individuals. The target of most of these antibodies is unknown. Antimyelin antibodies are present in only about 30–40% of cases of MS, and only a small subset of this fraction appear to be directed to an identified myelin antigen, MOG. It is evident that further work is necessary to define both the role and additional targets of antimyelin antibodies in MS, which is not an easy task in a disorder with apparent heterogeneous pathology and pathogenesis.

## Figures and Tables

**Table 1:** Induction of EAE or EAN and antimyelin antibodies by myelin antigens.

Antigens	EAE/EAN	Antibodies			
		Demyelinating		Myelination Inhibiting	
		CNS	PNS	CNS	PNS
CNS Antigens					
Whole CNS	+	+	-	+	-
MBP	+	-	-	-	ND
GC	-	+	+	+	+
M2	ND	+	ND	ND	ND
MAG	-	-	-	-	ND
PLP	+	-	-	-	ND
GM1	± ^a^	-	-	-	-
CMIP	± ^b^	+	-	+	ND
MOG	+	+	ND	ND	ND
PNS Antigens					
Whole PNS	+	±	+	+	+
P_2_ protein	±	-	-	-	-

ND: not determined; EAE: experimental allergic encephalomyelitis; EAN: experimental allergic neuritis; CNS: central nervous system; PNS: peripheral nervous system; MBP: myelin basic protein; GC: galactocerebroside; MAG: myelin-associated glycoprotein; PLP: proteolipid protein; CMIP: chloroform-methanol insoluble protein; MOG: myelin oligodendrocyte glycoprotein. ^a^ Several reports of induction of an experimental demyelinating disease in rabbits. ^b^ No clinical EAE but occasional inflammatory infiltrates histologically.
